# Approaches in cooling of resistive coil-based low-field Magnetic Resonance Imaging (MRI) systems for application in low resource settings

**DOI:** 10.1186/s42490-021-00048-6

**Published:** 2021-02-12

**Authors:** Faith Natukunda, Theodora M. Twongyirwe, Steven J. Schiff, Johnes Obungoloch

**Affiliations:** 1 Department of Biomedical Engineering, Mbarara University of Science and Technology, Mbarara, Uganda; 2 Department of Mechanical Engineering, Mbarara University of Science and Technology, Mbarara, Uganda; 3 Centre for Neural Engineering, Departments of Engineering Science and Mechanics, Neurosurgery, and Physics, The Pennsylvania State University, Pennsylvania, USA

**Keywords:** Low-field MRI, Resistive, Cooling, Temperature, Power dissipation

## Abstract

Magnetic Resonance Imaging (MRI), a non-invasive method for the diagnosis of diverse health conditions has experienced growing popularity over other imaging modalities like ultrasound and Computer Tomography. Initially, proof-of-concept and earlier MRI systems were based on resistive and permanent magnet technology. However, superconducting magnets have long held monopoly of the market for MRI systems with their high-field (HF) strength capability, although they present high construction, installation, and siting requirements. Such stringent prerequisites restrict their availability and use in low-middle income countries. Resistive coil-based magnet, albeit low-field (LF) in capacity, represent a plausible boost for the availability and use of MRI systems in resource constrained settings. These systems are characterized by low costs coupled with substantial image quality for diagnosis of some conditions such as hydrocephalus common is such regions. However, the nature of resistive coils causes them to heat up during operation, thus necessitating a dedicated cooling system to improve image quality and enhance system longevity. This paper explores a range of cooling methods as have been applied to resistive magnets, citing their pros and cons and areas for improvement.

## Background

The principles of Nuclear Magnetic Resonance have been applied long before the discovery of Magnetic Resonance Imaging (MRI). However, it was not until 1973, when Lauterbur [1] demonstrated image construction, that a rapid growth in Magnetic Resonance Imaging commenced. Magnetic resonance imaging has been of outstanding impact in the healthcare industry. As a non-invasive method, it has gained popularity over x-ray computer tomography in the diagnosis of the brain, spine, nervous and cardiovascular systems on account of its notable image quality especially for soft tissue imaging. MRI systems generally employ resistive, permanent, or superconducting magnets to produce a homogeneous magnetic field that uniformly polarizes the nuclear magnetic spins of the subject.

In 1980, the first commercial clinical MRI was made by Fonar. It was constructed using a heavy permanent magnet [2]. Permanent [3–5] and resistive [6, 7] magnets were the precursor technologies for the early commercial MRI scanners before superconductive magnets were discovered. Resistive magnets, although lighter than permanent magnets, exhibited power requirements as high as 40 kW of electrical power [8] and low field strengths, which often did not exceed 0.3 T before the power requirements made them uneconomical. Permanent magnets, although they were extremely heavy, could give fields up to 1 T [9–11] with very low power requirements. The advent of superconducting magnets and their proliferation, overtook their predecessors; initially with field strengths in the range of 0.35 to 0.6 T [12–14] and later 1 T and more. Superconducting magnets were considered lighter than permanent magnets and yet required no electrical power to maintain their magnetic field as did resistive magnets. Eventually, the 1.5 T commercial scanner became popular in 1985 and by 1998, the commercial 3 T imager [15] had started growing in market share.

Superconducting magnets offer higher signal-to-noise ratio, higher field strengths, higher image quality, lower lifecycle costs and generally shorter imaging times [2, 16, 17], albeit presenting very high capital costs. Permanent magnets have a fixed field and considerably low power requirements. They permit different configurations such as open-access MRI systems [3–5]; however, they are very bulky and heavy. Resistive magnets produce weaker fields, are less expensive [18] and exhibit smaller fringe fields than superconducting magnets owing to their low fields of operation. They have over the years sought a share of popularity in the commercial sphere, however, have been disadvantaged on several fronts as discussed in the next section.

In this work, we present the motivation for low-field magnets with specific focus on resistive conductors and why it is imperative to consider a thermal management system. We proceed by presenting methods that have been applied in thermal management over the years, their advantages, limitations as well as factors to consider when selecting one method over the other.

## Main text

### Materials and methods

An in-depth keyword-based search was conducted in academic databases such as Google Scholar, PubMed, Science Direct, Wiley, Springer and BioMed for publications on resistive magnets. Pertinent postgraduate theses were also included in the study.

#### Inclusion criteria

Studies that presented magnetic field strengths below 200 mT were considered and have been categorized as low-field (LF) magnetic field in this work. It is for such LF technologies that approaches to thermal management are regarded. Systems built from resistive conductor in the form of solid or hollow wire, thick strips of conductor as well as coils machined from resistive conductor material have been considered. Furthermore, the articles were required to have systems with successful application of magnetic resonance imaging or at least designed with intended application in the same.

#### Exclusion criteria

Articles that involved magnets requiring superconducting wire or cryocooling of any form have been excluded in this study. Bitter-type coils as well as hybrid coils, although presenting a resistive component have not been considered in this work. Moreover, studies involving resistive coils for gradient and radiofrequency systems were omitted in this work.

### Motivation

#### Motivation for use of LF MRI

LF MRI was the breakthrough point of MRI into the commercial arena, with the first commercial MR Imagers based on either permanent magnets or resistive electromagnets, however, high-field (HF) systems quickly ousted them with expensive superconducting magnets, thus alienating developing countries. LF MRI, considered an underdog to high field systems, is lately stirring keen interest in scientists. Resistive coil-based MRI systems exhibit flexible construction and siting requirements which makes them a tenable candidate for the spread of MRI systems in Low-Middle Income Countries (LMIC) where there are less than 0.1 Magnetic Resonance imagers available per 1,000,000 people [19]. Some of the reasons cited for poor distribution and utilization of MRI units in these countries include high acquisition, infrastructural and maintenance costs, and technical expertise needed to operate and use MRI systems [20, 21]. Geethanath and Vaughan [22] compared the number of MRI scanners against population, life expectancy, internet users and income group and demonstrated that resource constrained settings that have a very low number of MRI units also exhibited low life expectancy and low internet usage compared to more developed economies. This story can be changed by the adoption of LF MRI technologies. A recent review by Sarracanie and Salameh [23] cites several examples of LF MRI systems with magnetic field strengths from less the 1 mT to 199 mT that have given quality of images for clinical diagnosis.

Resistive LF MRI systems are substantially less expensive than HF MRI systems. The design, repair, and replacement of hardware in such systems is sustainable and inexpensive, and given their small footprint, siting requirements for the system are significantly reduced. Open MRI systems are possible with low field which makes them suitable for claustrophobic patients as well as neonatal patients who might need direct access during the imaging process [24]. Low field scanners eliminate the need for an imager to perform all MRI screenings often required with High Field systems. Dedicated scanners for specific body parts and of varying functionality and image quality are tenable with low field.

Superconducting magnets which require about 1700 l of liquid Helium [25] for operation, and increasing price and anticipated future shortages in availability of liquid Helium is creating an increasing demand for MRI magnets that are free of liquid Helium [26, 27]. LF MRI magnets have much lower cooling requirements than superconducting magnets. They are also more suitable for some NMR applications such as fast field cycling given that superconducting magnets give higher fields at the expense of slower switching and higher fabrication costs [28]. LF MRI systems allow for dedicated imaging of body parts which are small and not very easily explored with whole body scanning systems [29]. In such cases, the patient does not have to experience severe discomfort from claustrophobia in the whole-body systems. A review of commercial extremity LF MRI scanners is given by [30]. Hayashi et al. [31] and Konar and Lang [32] have made compelling arguments for the use of low field MR scanners.

#### Motivation for cooling

There is a renewed effort in the development of resistive coil based LF MRI systems, especially for applications in LMIC. Resistive wire based LF MRI machines are made by winding coils with many turns to generate required magnetic field for the system. During operation, current flows through the conductor wire which has a finite resistance. This resistance to the flow of current manifests as heat, also commonly known as joule losses. Conducting wire is often insulated to prevent short circuiting of the current. Magnetic field strength of a magnet depends on the current flowing through the conductor. The power deposited in a coil increases as the square of the field strength thus, maximum field strength is subject to the cooling ability of the coil.

A rise in temperature of the conducting wire, however, affects the insulation used during winding causing it to age drastically, making it the weakest link in the life of the coil [33]. Heating in a LF MRI increases the noise in the images produced and can also affect patient comfort [34, 35]. Because of increase in temperature above the thermal limits of the insulation, serious damage of the insulation can occur, and hence short circuiting between the coil conductor and surrounding equipment. The magnetic field in resistive coils is highly susceptible to small expansions. Macovski [36] and Kedzia [37] both observed a drop in magnetic field due to adjustment of the coil positions by the slight expansion from increase in temperature of a coil. Some methods have been proposed by various authors on minimization of temperature rise in resistive coils, the following section discusses this work.

### Cooling methods applied

Cooling systems can exhibit conductive, convective, or radiative modes of heat transfer. Often, a combination of these modes is experienced, although one of the modes generally exhibits greater dominance. Conductive cooling works by the transmission of heat through stationary matter; the two bodies across which heat is being transferred are in physical contact [38]. The matter is stationary on a macroscopic scale; however, on a microscopic scale, the atoms are vibrating and are in thermal motion. Convective heat transfer allows for the transfer of heat by macroscopic motion of a fluid. The convective mode of cooling can take the form of air cooling (natural or forced convection), or liquid cooling (water, oil, or any high thermal conductivity liquid and this can be by passive or active means) [39]. Heat transfer by radiation is independent of physical contact; it occurs by transmission of infrared electromagnetic waves through space. The following section discusses how different authors applied one or more heat transfer modes to their resistive coils. Table 1 presents a summary of the methods that have been applied to cool resistive magnets.

**Table 1:** A summary of cooling methods applied to resistive wire-based magnets and their challenges

Author(s)	Paper Title	Conductor Type	Cooling Mode	Cooling Medium	Closed/Open System	Cooling Type	Challenges/Future work
Gosselin & Bejan [40]	Constructal-theory Network of Conducting Paths for Cooling a Heat Generating Volume		Conduction	High thermal conductivity discs	N/A		Need for liquid cooling at higher field strength
Tsai et al. [41]	An Open-access, Very-low-field MRI System for Posture-dependent 3He Human Lung Imaging	Square solid copper wire	Convection	Air	Open		Need for liquid cooling at higher field strength
Gardner et al. [42]	Production of a Uniform Magnetic Field by Means of an End-Corrected Solenoid	Copper Ribbons	Convection	Water		Active/Forced	Eddy currents from Aluminum cylinder
Redpath et al. [43]	A Low Field NMR Imager for Clinical Use		Convection	Water	Open	Active/Forced	Use a closed system with recirculation of coolant
Lurie et al. [44]	Design, Construction and Use of a Large-sample Field-cycled PEDRI Imager	Copper sheets	Convection	Water		Active/Forced	Explore cooling in the axial direction
Sciandrone et al. [45]	Compact Low Field Magnetic Resonance Imaging Magnet: Design and Optimization	Copper tape	Convection	Water	Open	Active/Forced	Closed system with deionized water
Grafendorfer et al. [46]	A 0.2 T Homogeneous Resistive Knee Magnet for Remotely Polarized MRI	Solid copper wire	Convection	Water	Closed	Active/Forced	Explore hollow conductors
Gilbert et al. [47]	Design of Field-cycled Magnetic Resonance Systems for Small Animal Imaging		Convection	Water	Closed	Active/Forced	Decreased cooling efficiency from indirect contact of coil with coolant
Matter et al. [48]	Rapid Polarizing Field Cycling in Magnetic Resonance Imaging	Square hollow copper	Convection	Water		Active/Forced	Pressure drop and possible corrosion in fluid channel
Alford et al. [49]	Design and Construction of a Prototype High-Power Bo Insert Coil for Field-Cycled Imaging in Superconducting MRI Systems	Square solid copper wire	Convection	Water	Closed	Active/Forced	Direct cooling with deionized water for better cooling efficiency
Savukov et al. [50]	Non-cryogenic Anatomical imaging in Ultra-Low Field Regime: Hand MRI Demonstration		Convection	Air	Open		Need to use forced air cooling when operational current is increased
Busch [51]	Ultra-low Field MRI of Prostate Cancer using SQUID Detection	Litz wire	Convection	Liquid Nitrogen		Passive	Hotspots were formed
		Hollow copper tubes		Water		Active/Forced	Cool between pulses to minimize eddy currents
Lips et al. [52]	Magnet Design with High Bo Homogeneity for Fast-Field Cycling NMR Applications		Convection	Perfluoropolyether	Closed	Active/Forced	
Morgan et al. [53]	A Readout Magnet for Prepolarized MRI		Convection	Air		Passive	Need for forced cooling at higher magnetic strengths
Bidinosti et al. [54]	In Vivo NMR of Hyperpolarized 3He gas in Human Lung at Very Low Magnetic Fields	Solid copper wire	Convection	Air		Passive	Need for forced cooling at higher magnetic strengths
Sousa et al. [55]	Design of Nuclear Magnetic Resonance Fast Field Cycling Air Cored Magnet	Copper bar/wire	Convection	Transformer oil	Closed	Active/Forced	Heating of one end of coil due to direction of cooling.
Hilschenz et al. [56]	Remote detected Low-Field MRI using an optically pumped atomic magnetometer combined with a liquid cooled pre-polarization coil	Litz copper wire	Convection	Silicon Oil	Closed	Active/Forced	Low flowrate Highly viscous coolant
Lother et al. [57]	Design of a mobile, homogeneous, and efficient electromagnet with a large field of view for neonatal low-field MRI	Solid copper wire	Convection	Air	Open	Active	Insufficient for higher field strengths
Obungoloch [58]	Development of Ultra Low Field Magnetic Resonance Imaging for Diagnosis of Hydrocephalus in Developing Countries	Solid copper wire	Convection	Air		Passive	Need to apply forced cooling with increased currents

#### Conduction cooling

Bejan [59] exploited the idea of cooling by conduction by using a finite amount of high conductivity material to draw heat away from the source to the heat sink in cooling of electronics. Later, Gosselin and Bejan [40] applied a similar principle to a solenoid by inserting discs of high thermal conductivity material of determined thickness to separate the solenoid into sub-coils. Non-current transmitting discs were inserted to occupy a small fraction of total system length. Heat flowed axially through the windings and radially in the discs towards the heat sinks. More efficient cooling could be achieved with an increase in the amount of high conductivity material used. Radial heat transfer would be dominant even when the discs are carriers of cooling water. Furthermore, while studying the relationship between heat transfer and electromagnetic properties, it was observed that an electromagnetically optimized solenoid is smaller than one optimized both thermally and electromagnetically. A proposition into applying different cooling methods, assessing the mechanical integrity of the solenoid as well as minimizing costs in construction of such electromagnetic systems was made.

#### Air cooling

Tsai et al. [41] designed an open access exceptionally low field MRI system. The four coil biplanar magnet produced a magnetic field of 6.5 mT when a current of 42.2 A was supplied and produced approximately 4 kW of resistive heat particularly from the outer coils. The outer Bo coils of diameter 2.10 m were wound using AWG 6 square copper wire bound by high viscosity thermally conductive epoxy against an L-shaped aluminum channel to which a silver-based thermally conductive paste was applied to enhance conductive cooling. The inner Bo coils were wound and bound in the same way as the outer coils to Nylatron, which was mounted onto aluminum flanges. The aluminum channel functioned as a large heat sink; however, most of the cooling was done by convective air flow. Coil surface temperatures did not rise beyond 70 °C. The power system was cooled using a 2-kW recirculating liquid chiller to pump ethylene-glycol and water mixtures at 15 °C through two 0.5-in. copper tubes. The pumping was done continuously during imaging and the temperature of the cooling tubes did not exceed 40 °C.

In Non-cryogenic anatomical imaging in the ultra-low field regime: Hand MRI demonstration, Savukov et al. [50] demonstrated the use of atomic magnetometers with an ultra LF non-cryogenic scanner that was light weight, inexpensive and compact, without the need for shielding. The static magnetic field was achieved using a 4-coil Lee-Whiting configuration wound on a 33 cm diameter G-10 cylinder. A short-air cooled solenoid generated a prepolarizing field of 0.1 T. Although little detail is given on the design of the cooling system, the authors noted that the system was capable of running on twice the amount of current they used in the study and would therefore have to include a forced air cooling system to control thermal heating.

Lother et al. [57] also designed a mobile four coil resistive magnet wound with enameled copper wire and mounted on steel plates. The compact design allowed the fitting of uniquely designed gradient coils to each side for efficient space use. The magnet was designed for air cooling provided the power dissipated did not exceed 500 W when operating at 23 mT. Cooling efficiency was facilitated by steel plates that doubled as magnetic shields. Estimated power dissipation was calculated for 2 kW and 5 kW for field strengths of 50 mT and 80 mT respectively, which would have needed liquid cooling.

#### Liquid cooling

Gardner et al. [42] applied liquid cooling to a solenoid for application to beta-ray spectrometry. The 47.5 mT solenoid was wound using thin copper ribbons of width $1\frac{1}{4}$ in and thickness 0.021 in. The copper ribbons had a single layer of insulation facilitating heat transfer in the radial direction to the heat transfer plate. Heat transfer between the insulation and heat transfer plate was promoted by applying silicone Silastic to fill any air gaps, and the whole coil assembly mounted onto an aluminum cylinder. The thermal gradient was enhanced by running water in alternate directions through $\frac{3}{16}$ in copper tubes soldered to the heat transfer pipes. At full power (10 kW, 475 G), the temperature of the coils was found to be 5 °C above that of the cooling fluid. A thermal analysis of the cooling system was not presented, nor was there any comment on possible eddy currents from the aluminum cylinder or cooling system.

In ‘A Low Field NMR Imager for Clinical Use’, Redpath et al. [43] also applied water cooling to a four-coil air-cored resistive vertical field electromagnet with a central field of 0.08 T. The 1.9 m high and 2 tons magnet demanded 25 l/min of water to reduce temperature rise and an additional 7.5 l/min of water to cool the power supply. The cooling water was supplied from a header tank and was not recirculated in the system but run into the drain.

A combination of air and water cooling was, however, applied by Lurie et al. [44, 60], while designing and constructing a large Proton-electron Double-Resonance Imager. The primary magnet, a whole-body sized permanent magnet with a vertically oriented field of 59 mT, was cooled by blowing air from the room using a fan over the magnet’s thermal cover. Although the magnet had a large thermal time constant of a few days, cooling was done to obviate inhomogeneity in the magnetic field from thermal stresses. The room temperature was kept at 22 °C by an air conditioner. The secondary magnet was a resistive magnet of copper sheets wound in saddle configuration. It was water cooled on the outer surface of the cylinder that holds it; however, no additional information was shared on the construction and operation of the system.

Sciandrone et al. [45] designed and optimized a low field imaging magnet. The magnet, wound with copper tape, 6.3 mm wide and 2 in thick had a magnetic field strength of 0.1 T and power requirement of 2.2 kW. The eight coils that made up the magnet were mounted on a copper cylinder and kept in position by aluminum rings. The copper cylinder was cooled using ordinary water in an open circuit which confined the temperature rise of the copper cylinder to 40 °C. They recommended the use of deionized water in a closed-circuit cooling system.

Lips et al. [52] designed a fast field cycling magnet of magnetic field 0.95 T at 800 A of current. Six coaxially mounted layers of thick cylindrical coils of varying coil width were cooled radially with perfluoropolyether flowing from the top and bottom on the inside of the inner layer to the outside of the windings. The flow was turbulent and directed in such a way that it was strongest at the points where the windings were thickest. No significant heat gradient was observed inside the coil in the axial direction. Water in an internal circuit was used to cool the coolant, maintaining coolant temperature of about 12 °C via a 50-kW plate heat exchanger while conductor temperature was on average 26 °C.

Moreover, Grafendorfer et al., [46] designed a four-coil homogeneous 0.2 T water cooled resistive knee magnet, with water carrying copper tubes sandwiched between cooling plates attached to the coil. The cooling plates were split every 22.5 degrees to minimize losses from eddy currents. The temperature rise at the coil’s hottest spots capped at 40 °C when the chilled water flowed at 15.14 l/min. The power dissipated was 17.5 kW. Hollow conducting wire was proposed for enhanced thermal conductivity.

A detailed construction of a LF MRI cooling system has been given by Gilbert et al. [47]. Forced water aluminum cooling plates were used for heat transfer in their field cycling system for imaging small animals. The cooling plates were placed at each face of each coil of the main magnet and were sandwiched within the polarizing magnet as well as at its ends. A radial gap of about 7 cm was allowed between the polarizing and the readout magnets for cooling tubes and support systems. In [61], two thermal models were developed to assess the thermal performance of the magnets; equilibrium and dynamic models which they validated by experiment. It was observed that the temperatures of the polarizing magnet were limited to 150 °C and those of the readout magnet rose only slightly owing to the forced water cooling and a low duty cycle of 10%. A flow rate of 9 l/min was used for both the readout and polarizing magnets. Both magnets were driven by a continuous current of 100A and the temperature rise did not exceed 20 °C during a 5-min scan period [62]. They also noted that the time between successive operations of the system was the most important cooling parameter in thermal performance. There was generally a good correlation between the dynamic model and the experiment, although oversimplification of the models could have caused the divergence in some values [61].

In contrast, Matter et al. [48] used square hollow conductor wire in construction of a LF pre-polarized MRI system. The coil, 12.7 cm inner diameter was designed to image a human wrist. The polarizing coil, a segmented solenoid connected in series was water cooled. The cooling fluid was passed through the conductor as a means of removing heat from the system, however, to increase heat transfer, the water channels were connected in parallel. This allowed them to achieve currents as high as 200 A and a field strength of 0.42 T. The system efficiently removed 16 kW dissipated as heat from the coils. It was purported that with water-cooling, polarizing fields as high as 1 T are plausible.

Sousa et al. [55] designed a 1.6 T Fast Field Cycling Air Cored Magnet for NMR. The magnet was optimized for maximum current density, minimum power losses and minimum volume. Driven by a current of 200 A, 78.4 kW of power was dissipated, however, cooling layers were optimized within the design to ensure efficient cooling with the coils occupying minimal volume. Transformer oil flowed through the hydraulic layers as a coolant from one end of the coil to the other through the full length of the coil [63].

Alford et al. [49] designed a Bo insert coil for use in a superconducting MRI. The solenoid coil was wound with AWG4 square cross-section wire, reinforced with epoxy. One hundred meters of 6.35 mm OD thin-walled Teflon tubing was wound proximal to the inner and outer surfaces of the magnets for cooling of both the primary and shield (108 turns AWG7 Litz rectangular cross section wire) magnets. Thermal conductivity was facilitated by potting the whole assembly in thermally conductive epoxy resin (part #51–3100). Observation during testing showed relatively poor conductivity across the Teflon cooling tubes; thus, the limiting factor to cooling performance. A thermal resistance of 0.0175 °C/W was measured for a supply of 6 l/min of chilled water for cooling. It was proposed that the performance of the insert be made better by replacement of the Teflon cooling tubes with direct cooling using deionized water to enhance cooling efficiency.

Busch [51] used ultra-low field MRI for prostate cancer measurements. The static field magnet was a biplanar four coil magnet although only the larger coil of diameter 1.37 m and 40 turns was implemented. The initial pre-polarizing coil was wound from Litz wire and cooled by liquid nitrogen via special cooling channels designed within the coil. Cooling of the coil was only done until an overall resistance of 1.4 Ω was reached, although the resistance would rise to as high as 2.5 Ω. During operation, cooling would be discontinued to avert instauration of hotspots from uneven cooling which could affect insulation of the wire at uncooled locations. Cooling would however be done just before imaging to thermalize the wire. An attempt to cool the Litz wire with water decreased the self-resonant frequency of the coil given that the high dielectric constant of water substantially increased the turn-turn capacitance of the coil.

A decision to change the pre-polarizing coil to allow for higher current densities and magnetic field resulted in the use of hollow wire conductors with provision for water cooling. The new coil consisted of 240 turns and produced a field of 150 mT at 200 A. The coil dissipated 10 kW of heat and required 4 l/min of cooling water to keep the temperature rise below 40 °C at 50% duty cycle. Heat dissipation would rise to as high as 20 kw when the coil was powered at 200A. However, the water-cooled coil caused eddy currents to flow around the aluminum shielded room that was made from 6.4 mm thick Aluminum plates. A decrease in the thickness of the Aluminum shield minimized this noise. During imaging of a phantoms at 120 mT, a waiting time of 1.4 h was allowed with in the total scanning time of 2.4 h so the coil could cool between pulses, although a higher flow rate would decrease and possibly eliminate this time [51].

Hilschenz et al. [56] however registered a different experience with their Litz wire pre-polarizing coil. The coil was wound from a 3.6 mm diameter, 19 strands Litz copper wire. The 300 turns were arranged in 10 layers and immersed in silicon oil as coolant, enclosed within an acrylic chassis. The coil was wound in a manner that permitted insertion of carbon bars as spacers for coolant to flow between every layer. This considerably improved cooling efficiency. The use of silicon oil inhibited sparks from the high voltages for an increased switch off rate. With the coil running at 30 A, 3 s on and 1 s off and a duty cycle of 75%, the temperature of the coils did not exceed 40 °C. The pressure pump used to circulate coolant worked at 90% of its power limit, albeit the flow rates of coolant were a limiting factor due to its high viscosity. Given the magnetic field from Bp coils need not be homogeneous, designing of the coil with intermittent spacers may be acceptable for such a system. However, it is fundamental to assess the effect of the spacers on homogeneity of the field strength when such a design approach is applied to static magnetic field coils.

#### No cooling

Moreover, Morgan et al. [53] proposed the application of no cooling while designing a readout magnet for Pre-polarizing MRI. A comparison of two designs based on an equation to minimize temperature rise in the system was made under the assumption of an exceptionally large thermal time constant to exclude forced cooling. The Helmholtz system was bulky and of a high-power requirement, implying a possible need for cooling. The power requirements and heating load of the system could be decreased by adding weight to an already heavy system. The six-coil system, however, was designed for a magnetic field of 0.07 T and dissipated 6.7 kW of power at 100 A. After homogenizing the field, applying an exceptionally large thermal time constant to estimate the cross-sectional area of the coils such that the temperature rise was limited to a set amount, permitted elimination of active cooling of the system altogether. A slow heating rate of 6 °C/min was applied during design of the coils and at 1 MHz and a current of 12.7A, a temperature rise of 20 °C was expected over a period of 6 min. A temperature rise of 200 °C would be expected in the same time period at 3 MHz, thus addition of forced cooling by air or water to minimize temperature rise in the conductors would be advantageous.

Bidinosti et al. [54] in their work on studying human lungs at very low magnetic fields implemented a magnet of seven co-axial coils of 144 turns each wound from copper wire 2 mm in diameter. The coils were wound on an aluminum annulus and required no forced cooling at the desired magnetic field of 6 mT driven by a current of 8.7A and 940 W of power. The low magnetic field made active cooling unnecessary.

Obungoloch [58] developed an Ultra LF MRI for diagnosis of hydrocephalus. The system consisted of a static magnetic field coil (Bm coil) operated at 2.66 mT, a prepolarizing coil, (Bp coil), Radiofrequency and gradient coils. The Bp coil, a solenoid constructed from enameled copper wire AWG 10 had a total of 1140 turns and gave a magnetic field strength per unit current of 5 mT/A at the center of the coil when supplied with a current of 10 A. During testing of the system, over a period of 90 min, temperature of the imaging volume and the Bp coil were simultaneously measured. A relatively constant temperature was measured in the imaging volume while that of the coil increased considerably by as much as 80 °C, thus a proposition to use fans to keep temperature rise manageable. The system had a combined power utilization of 550 W.

## Discussion and conclusions

### Discussion

The choice of cooling system for use in an MRI machine depends on the magnetic field strength requirement of the magnet, which is dependent on the material of the magnet. Most LF resistive magnets are built from three common methods: (1) enameled solid wire, (2) hollow copper wire or (3) tape conductors. Each method has its own advantages and challenges to cooling. In case (1), cooling is often done at the surface of the coil; heat is transferred to the cooling surface via an enamel insulator which in many cases is the limiting factor. The likelihood of creation of hotspots in such a coil is remarkably high due to the creation of a thermal gradient from slow heat transfer via the insulation. Solid conducting wire was applied by [41, 49, 55, 63]. Hollow conductors (2) have been used by [48, 51]. Cooling is done by pumping the coolant through the center of the conductor; there are no thermal barriers because direct cooling is used, however, the costs of running such a system are high resulting from the hydraulic requirements for coolant circulation. The third case (3) was applied by [44, 45, 60]. The coils are surface cooled at their faces or edges, and present better heat transfer than (1) because the insulation is only between layers. The magnetic field strength of a magnet depends on the current flowing through the conductor, yet the power deposited in a coil increases as the square of the field strength, therefore, the maximum field strength is tied to the ability to cool the coil.

In many of the systems reviewed, addition of a cooling system was a necessity except with [53] who applied a slow heating rate to eliminate the need for cooling and [54, 58] who were working with sufficiently low magnetic fields and currents that allowed passive convection without overheating of the coils. Conduction based cooling [40] and air cooling [41, 57] have been applied in situations where the heat load was low enough not to cause destruction of the conductor. However, it ceases to be effective when magnetic field requirements are scaled up, thus, necessitating a form of liquid cooling. The use of conductive or air cooling is often limited to heat transfer at the outermost surfaces of the magnet. This can be overcome by modifying a conduction-based cooling system by adding strips of high conductivity material between coil layers during winding that will transfer heat to the discs between the coils. The surface area over which air cooling is possible can be increased by adding spacers between subsequent layers thus availing more channels for air flow within the coil. These modifications introduce new challenges, and such include decreased field homogeneity, the need to use more conducting wire or strips, increased system weight. Table 2 is a summary of the advantages and limitations of the cooling systems reviewed in this work.

**Table 2:** Advantages and limitations of different cooling methods

Cooling Method	Advantages	Limitations
Conduction	• Good with low current density systems. • No moving parts required. • Can be coupled with convective cooling methods.	• There is a limit to how much heat transfer material is to be used. • Not suitable for high current density values. • Significant increase in system weight if more heat conductive material is added. • Eddy currents in case of excess material [42] and from changing fields
Air cooling	• Cooling medium is readily available. • Can easily be coupled with conductive cooling and indirect liquid cooling. • Dry method hence no fear of shocks from leakage.	• Limited to current densities of 2 A/mm^2^. • Low coefficient of heat transfer (between 2.5–10 (natural convection) 10–500 W/m^2^K (forced convection) [64]). • Often bulky with high space requirements.
Liquid cooling	• Relatively high coefficient of heat transfer (100–15,000 W/m^2^K [64]) • Compact design is possible hence lower space requirements. • No thermal barriers when direct cooling through hollow conductors is used	• Possible electric shocks in case of leakages • Corrosion of cooling channels due to high flow rate and pressure requirements • Additional power and operational costs from pumping

The use of liquid cooling excels above conductive and air cooling as an effective method of heat removal [51, 52, 55, 56, 63]. Authors such as [42–48, 51, 56, 60, 62] used water, whereas liquid nitrogen [51], perfluoropolyether [52], transformer oil [55, 63] and silicon oil [56] have also been used as coolant. Liquid cooling can either be applied in a closed or open loop cooling system. In an open loop system as in [45], coolant drains out during operation. This system is less expensive, easier to construct and has lower power requirements. Raw water is often used owing to its ready availability. In closed cooling systems, the coolant is recirculated through the cooling channels after its heat is removed by another fluid via a heat exchanger. Liquids including water can be used as coolant [52, 55, 56, 63]. Closed systems permit the use of other coolants that are more efficient and have milder effects on the coil than water, although they present an environmental risk in the event of leakage and eventual disposal at the end of the working life of the system. The use of ordinary water in such systems can eventually cause corrosion especially in hollow conductors due to the high hydraulic requirements and potential shocks and sparking for coil cases (1) or (3) if there is a leakage.

The decision on the location of cooling pathway and channels is as essential in the management of temperature rise as the choice of cooling method and medium. The resolution to select direct cooling over indirect cooling hinges on different factors. This decision is often unique to the magnet requirements. However, for any magnet design, in addition to the strength of the magnetic field, the following should be considered. 1) The coil configuration; the arrangement of coils to create the desired magnetic field is fundamental to the decision on a cooling system. Some configurations such as Lee-Whiting [65], Merritt [66] have significant separation between the coils which increases the surface area available for cooling compared with coil size. Long and thick solenoids are more challenging to cool. 2) The application of the system; the system can be a whole body or dedicated imager. It can be fixed or movable or required to work in a constrained environment or the field. A portable system will require a compact thermal management system, which may not be as critical for a stationary system. 3) The demand on the system during operation; this includes the number of hours of operation and the time allowed between screenings. Longer hours of operation and shorts intervals between screenings place high demands on the cooling system. 4) The space and weight requirements include the amount of space available for the system and its components and the maximum weight the system should have. When more space is available for the imaging system, a larger cooling system that could be less financially demanding can be considered. Available space can also mean that the volume of the magnet(s) can be larger, therefore, conductors with lower resistance can be used. This will decrease the thermal management requirements. 5) The energy cost of operating a cooling system should be considered. LMIC settings with low access to electricity and high electricity tariffs can benefit from systems with low running costs.

Therefore, one approach towards improving thermal management would be to design short and thick coils with edge cooling given that the distance over which heat transfer to the cooling channels will be short. Radial cooling of such coils is possible; however, the coils end up thicker. Consideration of coil configurations such as Helmholtz, Lee-Whiting and Merrit designs give considerably uniform field homogeneity and allow for short and thick designs. Long and thick coils can also be considered especially when the field requirements are high. However, it would be necessary to split the coil, preferably in the axial direction to provide short distances over which heat transfer can occur.

### Conclusion

The recent increased interest in low field magnetic resonance imaging necessitates critical consideration of the hardware limitations to its growth. Resistive magnets launched superconducting magnets that eventually overtook them for diverse reasons. One worth noting is their low fields and yet high-power requirements, hence the need for cooling. Several approaches to thermal management of LF MRI systems have been reviewed in this work. The decision on which method of cooling to apply depends on various factors, especially the design and construction of the magnet. Great costs are incurred when the incorporation of a cooling system is an afterthought or inadequate to meet the operational need of the magnet. Therefore, anticipation of future magnetic and cooling requirements without the need to redesign the cooling system or use unanticipated energy to operate the entire system is essential right from the design of the magnet.

## Data Availability

Not applicable.

## Abbreviations

AWG: American Wire Gauge
HF: High-Field
LF: Low-Field
LMIC: Low-Middle Income Country
MRI: Magnetic Resonance Imaging
NMR: Nuclear Magnetic Resonance
OD: Outer Diameter
